# How Nature Makes O_2_: an Electronic Level Mechanism for Water Oxidation in Photosynthesis

**DOI:** 10.1021/acs.jpcb.2c06374

**Published:** 2022-10-07

**Authors:** Felix Rummel, Patrick J. O’Malley

**Affiliations:** Department of Chemistry, School of Natural Sciences, The University of Manchester, ManchesterM13 9PL, U.K.

## Abstract

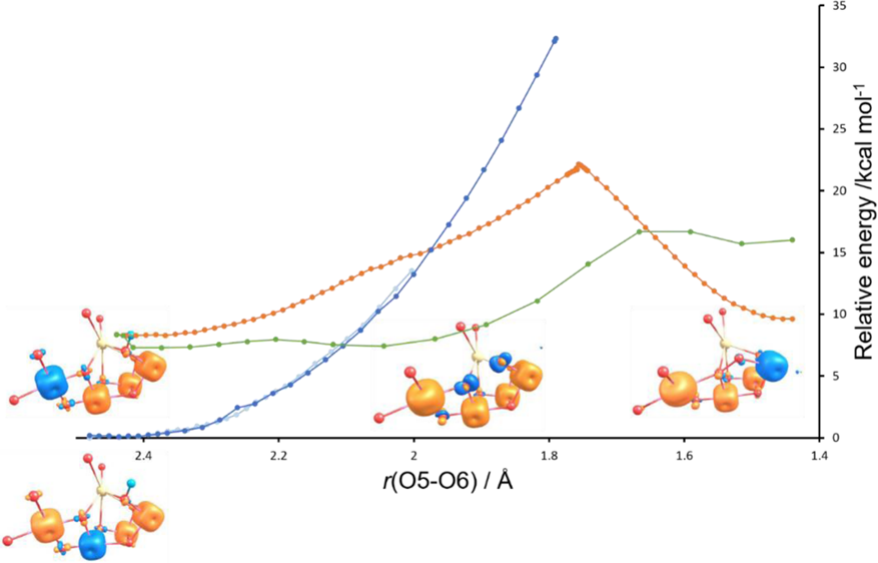

In this paper, we combine broken symmetry density functional calculations and electron paramagnetic resonance analysis to obtain the electronic structure of the penultimate S_3_ state of nature’s water-oxidizing complex and determine the electronic pathway of O–O bond formation. Analysis of the electronic structure changes along the reaction path shows that two spin crossovers, facilitated by the geometry and magnetism of the water-oxidizing complex, are used to provide a unique low-energy pathway. The pathway is facilitated via the formation and stabilization of the [O_2_]^3–^ ion. This ion is formed between ligated deprotonated substrate waters, O5 and O6, and is stabilized by antiferromagnetic interaction with the Mn ions of the complex. Combining the computational, crystallographic, and spectroscopic data, we show that an equilibrium exists between the O5 oxo and O6 hydroxo forms with an *S* = 3 spin state and a deprotonated O6 form containing a two-center one-electron bond in [O5O6]^3–^ which we identify as the form detected using crystallography. This form corresponds to an *S* = 6 spin state which we demonstrate gives rise to a low-intensity EPR spectrum compared with the accompanying *S* = 3 state, making its detection via EPR difficult and overshadowed by the *S* = 3 form. Simulations using 70% of the *S* = 6 component give rise to a superior fit to the experimental W-band EPR spectral envelope compared with an *S* = 3 only form. Analyses of the most recent X-ray emission spectroscopy first moment changes for solution and time-resolved crystal data are also shown to support the model. The computational, crystallographic, and spectroscopic data are shown to coalesce to the same picture of a predominant *S* = 6 species containing the first one-electron oxidation product of two water molecules, that is, [O5O6]^3–^. Progression of this form to the two-electron-oxidized peroxo and three-electron-oxidized superoxo forms, leading eventually to the evolution of triplet O_2_, is proposed to be the pathway nature adopts to oxidize water. The study reveals the key electronic, magnetic, and structural design features of nature’s catalyst which facilitates water oxidation to O_2_ under ambient conditions.

## Introduction

Every oxygen molecule we breathe is produced from two water molecules in the photosystem II protein complex of higher plants, algae, and cyanobacteria. This highly endothermic reaction is carried out during photosynthesis using visible light energy under ambient conditions. To perform this task, a unique water-oxidizing catalytic complex, Mn_4_CaO_5/6_, evolved some 3 billion years ago. This complex oxidizes two water molecules to molecular oxygen at a rate approaching 1000 s^–1^ at ambient temperature and pressure.^1,2^ Besides being one of the most important reactions in biology, it is also of intense interest from a green energy perspective, where it is recognized to be the main barrier to the development of commercial solar devices for the generation of hydrogen from water.^3^ Water oxidation to dioxygen is challenging due to the high endergonicity (*E*° = 0.82 V (vs NHE) at pH 7) of the reaction and the associated need to remove four protons and four electrons with the formation of an oxygen–oxygen, O–O, bond. Two broad mechanistic proposals, water nucleophilic attack of metal oxo and direct metal oxo radical coupling, have been proposed for artificial water oxidation catalysis (WOC).^4^ Somewhat similar proposals have been put forward for WOC, namely water nucleophilic attack^5^ or oxyl radical–oxo coupling^6^ These require the generation of a reactive oxo species in the final Kok cycle S_4_ state. Artificial catalysts generally use very high-strength oxidizing agents to generate reactive oxo species, either radical oxygen species or highly charged metal electrophilic species. WOC on the other hand is limited to the approximately 1 V oxidizing power of the nearby tyrosyl radical, Y_Z_^OX^.^7^ The current mechanisms for WOC which propose the generation of a reactive “hot” oxo species in the S_4_ state need to explain how such a species can be generated when the oxidizing capability from the visible light energy available via the S_3_Y_Z_^ox^ oxidant is around 1V. It is also unclear how triplet O_2_ can be produced from the peroxo form with such a mechanism given that the last oxidizing equivalent has been used. 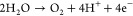


**Figure 1:**
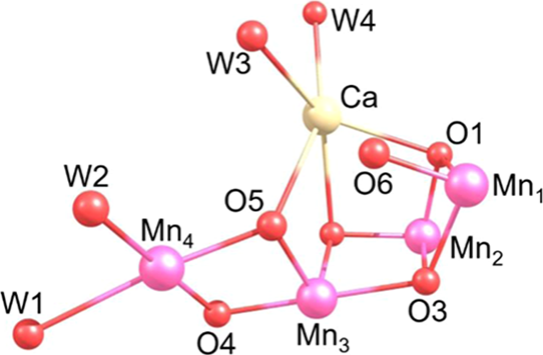
WOC catalyst core structure with orientation and numbering scheme used throughout.

An alternative mechanistic scenario is the dynamic equilibrium model of S_3_ speculated by Renger,^1^ consisting of a concerted reduction of Mn coupled to O–O bond formation.^8,9^ Such a mechanism would aesthetically require the WOC cluster design, Figure 1, to facilitate the lowering of the O–O bond formation barrier, permitting it to be readily transversed at room temperature without the generation of a reactive oxo form.

For the four-electron oxidation of two water molecules in the aqueous phase,^1^Figure S2, it is the first one-electron oxidation of water to form an oxyl radical that represents the major energy barrier with a reduction potential ≥2 V, well in excess of the 1 V available in S_3_Y_Z_^OX^. If the WOC can reduce this barrier, then the sequential four-electron oxidation of water is thermodynamically feasible with visible light energy. Here, we demonstrate that the WOC is designed to achieve this task by stabilizing the one-electron oxidation product of water as an [O_2_]^3–^ ion. Partial O–O bond formation and stabilization of this species are brought about by the unique architecture and magnetism of WOC, which facilitate the electron rearrangement between the O5 and O6 oxo forms engaged in O–O bond formation and the Mn1 and Mn4 ions of the WOC. This is combined with the stabilising antiferromagnetic alignment of the Mn1,3,4 ions with the unpaired electron of [O5O6]^3–^. This stabilizes nascent O–O bond formation in the S_3_ state, permitting low-barrier O–O bond formation, and is supported by the XFEL structural crystallographic data and by the EPR spectra obtained on the 2-flash state of the WOC.

## Results and Discussion

### Electronic Structure Analysis

**Figure 2:**
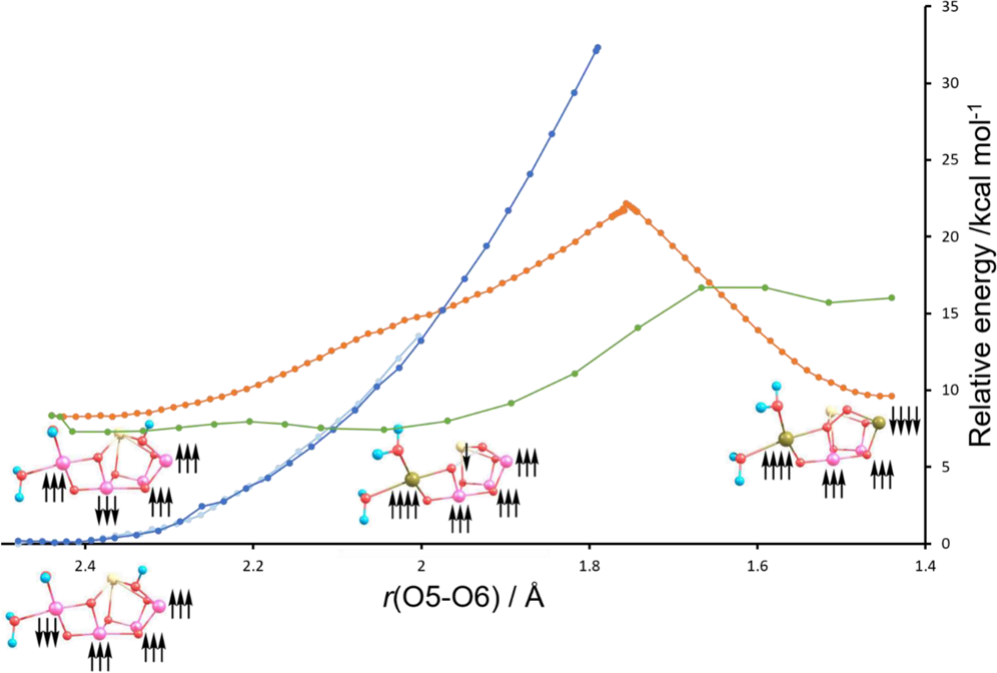
S_3_ state potential energy scans (PES) for oxo–hydroxo (Mn3 flipped, dark blue; Mn4 flipped, light blue), oxo–oxo (Ms = 3, red), and oxo–oxo (Ms = 6, green) forms. The spin alignments for the local energy minima along the PES are illustrated.

Our starting point on the pathway to O5–O6 bond formation is an O5 oxo–O6 hydroxo form of the WOC complex, Figure 1, formed after the initial formation of the S_3_ state. This corresponds to the *S* = 3 form detected by EPR with four Mn (IV) ions.^10^ O6 corresponds to the new oxygen atom detected by XFEL after the second flash.^11,12^ For this oxo–hydroxo model, seven broken symmetry, Ms, states are possible at the optimized geometry. We have shown^10^ that two of these, both *M*s = 3, [Mn4(↓↓↓)Mn3(↑↑↑)Mn2(↑↑↑)Mn1(↑↑↑)] and [Mn4(↑↑↑)Mn3(↓↓↓)Mn2(↑↑↑)Mn1(↑↑↑)], are the lowest in energy and govern the spin density of the complex, resulting in a spin distribution of close to 0.5, 0.5, 0, 0 and 0.0 for Mn1–Mn4. This explains the set of two large (Mn1 and Mn2)- and two small (Mn3 and Mn4)-magnitude ^55^Mn hfcs observed using EDNMR spectroscopy.^10,13^ Deprotonation of O6 leads to an O5 oxo-O6 oxo form. At the optimized geometry, two low-energy BS states are found, an Ms = 3 form [Mn4(↑↑↑)Mn3(↑↑↑)Mn2(↑↑↑)Mn1(↓↓↓)] and an Ms = 6 form [Mn4(↑↑↑)Mn3(↑↑↑)Mn2(↑↑↑)Mn1(↑↑↑)]. The energies of these oxo–hydroxo and oxo–oxo states are plotted as a function of O5–O6 distance in Figure 2. The oxo–hydroxo form is the lowest energy form for O5–O6 distances of 2.5–2.1 Å.

**Figure 3:**
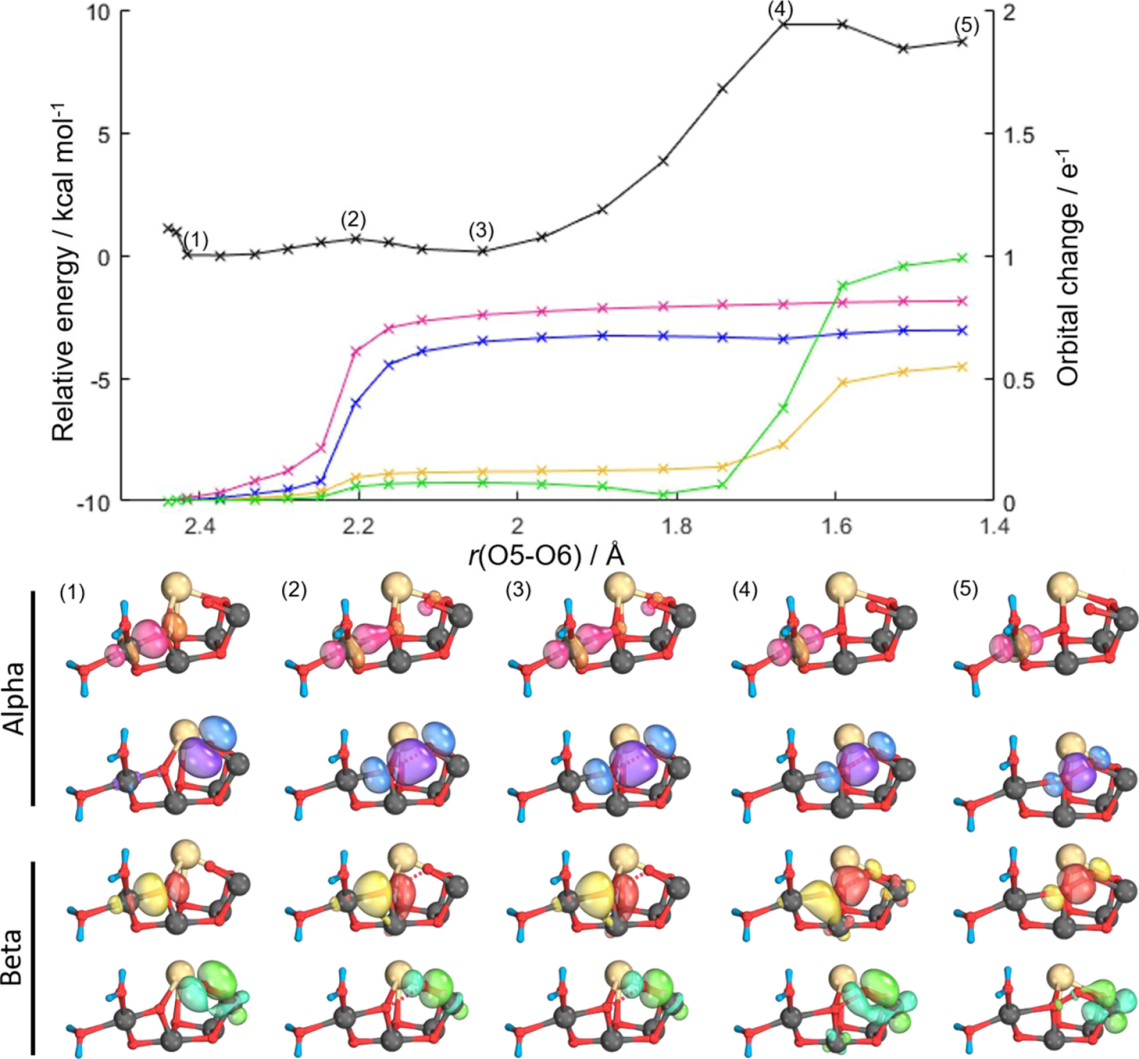
Intrinsic bond orbital (IBO) analysis of the Ms = 6 state of the oxo–oxo form. Top, potential energy surface (PES) for O5–O6 bond formation (black) accompanying IBO changes color-coded by the orbitals shown beneath. Representative IBOs are given at the points labeled on the PES above showing α and β spin evolution; see text for details.

**Figure 4:**
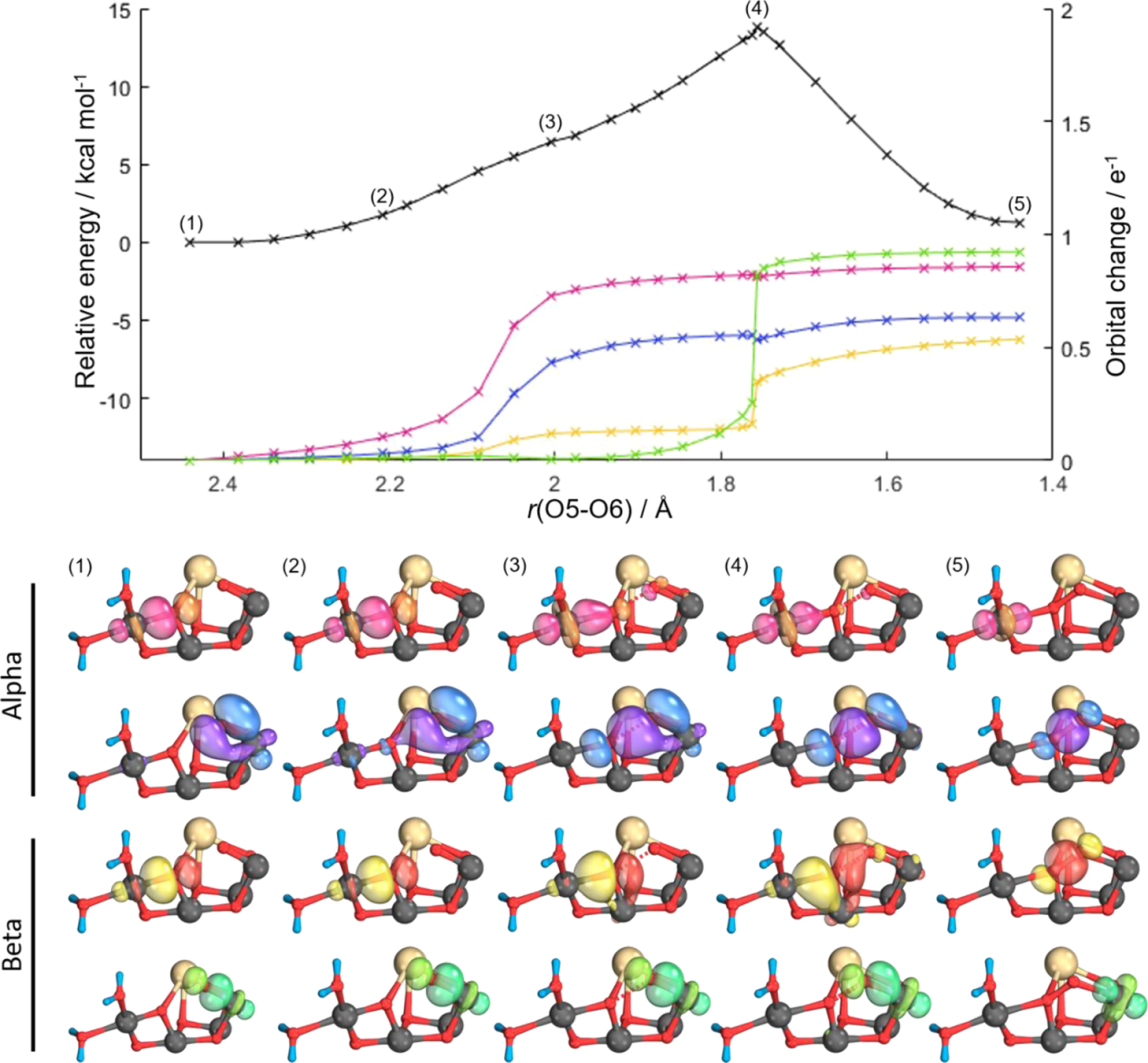
Intrinsic bond orbital (IBO) analysis of the Ms = 3 state of the oxo–oxo form. Top, potential energy surface (PES) for O5–O6 bond formation (black) accompanying IBO changes color-coded by the orbitals shown beneath. Representative IBOs are given at the points labeled on the PES above showing α and β spin evolution; see text for details.

At 2.1 Å, a spin crossover to the Ms = 6 oxo–oxo form is indicated. For the Ms = 3 oxo–oxo form, the crossover with the oxo–hydroxo PES occurs at a higher energy at an O5–O6 distance of 2.0 Å. The Ms = 6 state remains the lowest energy form up to an O5–O6 distance of 1.65 Å where a spin crossover to the Ms = 3 state occurs as peroxo is formed. At O5–O6 distances less than 2.0 Å, the oxo–hydroxo form becomes unstable, and convergence is not achievable. The PES scan in Figure 2 shows that two spin crossovers, facilitated by the unique geometry and magnetism of the WOC complex, provide a low-energy pathway for O5–O6 peroxo bond formation. To monitor the changes in electronic configuration and rationalize the relative energies of the different BS states as we traverse the PES, we monitor the changes in the intrinsic localized bond orbitals involved. These changes are demonstrated for the oxo–oxo Ms = 6 and the oxo–oxo Ms = 3 states in Figures 3 and 4, respectively.

For the oxo–hydroxo form, no changes are found in the IBOs in the region of 2.5–2.0 Å, and as mentioned above, this model becomes unstable at bond distances less than 2.0 Å. By contrast, significant changes are observed for both oxo–oxo forms. The IBOs which undergo significant changes are located by monitoring the root-mean-square deviation of every IBO from the initial partial charge distribution along the PES.^14,15^Figures 3 and 4 identify four main IBOs participating in bond-making and bond-breaking during the reaction. These are the α and β spin orbitals of the Mn_4_–O5 σ-bond, the β spin orbital of the Mn_1_–O6 σ-bond, and the α spin orbital of one of the π-bonding lone-pair orbitals on O6. As the O5–O6 bond distance is decreased from the nonbonded oxo–oxo form, the α electron density of the Mn_4_–O5 σ-bond evolves into a dz^2^ orbital on Mn_4_, Figures 3 and 4 (pink), at an O5–O6 bond distance of around 2.2 Å for Ms = 6 and 2.0 Å for Ms = 3. Concurrently, with this electron density rearrangement, the α density of the π-lone pair on O6 evolves to a σ-bond between the O6 and O5 oxygens, Figures 3 and 4 (blue). A Mayer bond order analysis,^16^Figure S3, also illustrates such a change with a decrease in the Mn_4_–O5 bond order from near 1.0 to near 0.5 and an increase in the O5–O6 bond order from 0.0 to near 0.4. In a similar fashion, Mulliken spin population analysis, Figure S4, shows a change in the spin population of Mn_4_ from near 3.0 to 4.0, signaling a reduction from Mn_4_(IV) to Mn_4_(III). For the Ms = 3 state, further progression along the PES shows that the β-electron of the Mn_4_–O5 σ-bond evolves to become the β-component of the O5–O6 σ-bond, Figure 4 (yellow), and the Mn_1_–O6 σ-bond β-electron density evolves into a dz^2^ orbital on Mn_1_, Figure 4 (green). For Ms = 6, the β-electron of the Mn_4_–O5 σ-bond again evolves to become the β-component of the O5–O6 σ-bond, Figure 3 (yellow), while in this case, a Mn_1_–O6 π-bond β-electron density evolves into a dπ orbital on Mn_1_, Figure 3 (green). Mulliken spin populations, Figure S4, correspondingly show an increase in spin population from 3 to 4 for Mn1, illustrating the reduction of Mn1 to high-spin Mn(III) for Ms = 3, whereas for Ms = 6, the electron transfer of a β-electron to Mn1 results in the occupation of a dπ orbital, Figure 3 (green), resulting in a spin population of 2 and corresponding to a low-spin form of Mn(III). The Mayer bond order values for both Ms states of Figure S3 show an increase in the O5–O6 bond order to near 1.0 as the peroxo is formed.

**Figure 5:**
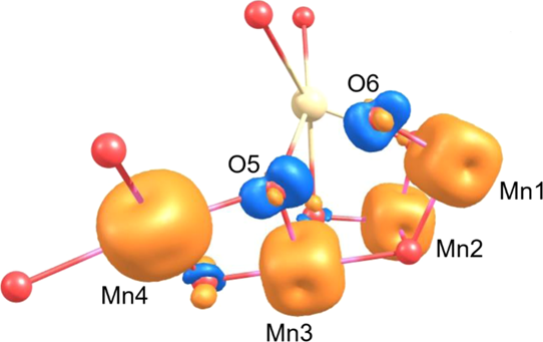
Spin density contour plot for the Ms = 6 oxo–oxo model at 2.0 Å on the PES, demonstrating the distribution of negative spin density (blue) on both O5 and O6 positions and signifying the presence of [O5O6]^3–^

Our key finding is that for the Ms = 6 oxo–oxo form, an electronic state corresponding to Mn4(↑↑↑↑)Mn3(↑↑↑)Mn2(↑↑↑)Mn1(↑↑↑)[O5O6](↓) is found as a shallow local minimum at an O5–O6 distance of 2.0 Å. The IBOs, Figure 3, show that electron movement has occurred from O5 to Mn4, leading to a high-spin Mn4 (III) and the formation of a nascent two-center one-electron O5–O6 bond. This species was identified previously by us^17^ as a shoulder on the Ms = 3 state (see Figure 4). While a shoulder on the Ms = 3 PES, it corresponds to a broad minimum energy structure on the Ms = 6 surface due to the favorable antiferromagnetic coupling with all four Mn ions. We note that this species has been referred by us^17^ and others^18,19^ previously as an O5 oxo–O6 oxyl form, but it is best and more appropriately described as [O5O6]^3–^ as a negative spin density is present on both O5 and O6, clearly demonstrated by the spin density plot for this form in Figure 5.

**Figure 6:**
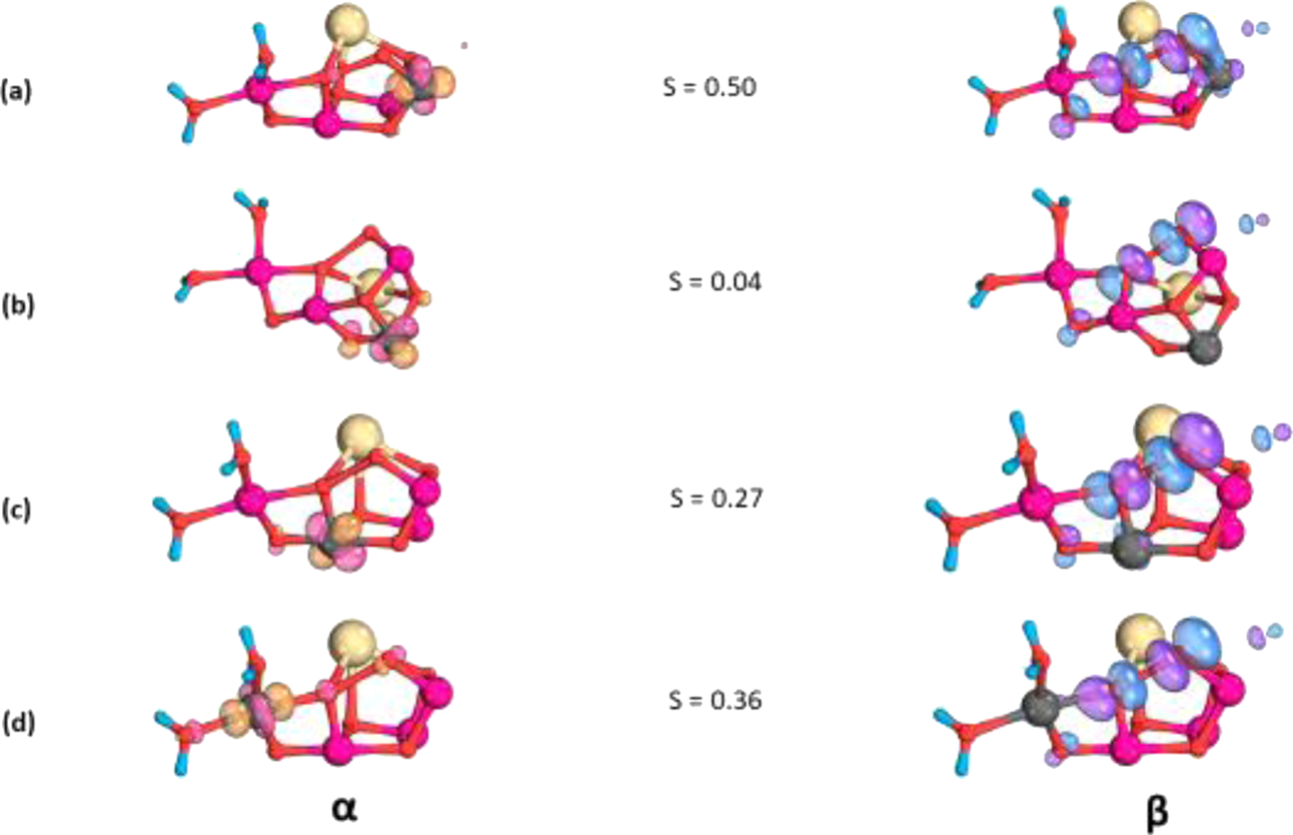
Corresponding magnetic orbitals for (a) Mn1–O5O6, (b) Mn2–O5O6, (c) Mn3–O5O6, and (d) Mn4–O5O6.

This Ms = 6 state is stabilized by the strong antiferromagnetic interaction occurring between the β-electron density shared between O5 and O6 and the α-electron spins on the Mn1, Mn3, and Mn4 ions. The strength of this antiferromagnetic coupling is quantitatively demonstrated by the large-magnitude O5O6Mn1, O5O6/Mn3, and O5O6/Mn4 *J* values calculated for this electronic arrangement (see Table S1) and also graphically illustrated by the large overlap integral value *S* calculated for the corresponding locally transformed Mn and O5O6 magnetic orbitals in Figure 6. The overlap integral value *S* is a measure of the strength of the orbital overlap and associated antiferromagnetic coupling between the magnetic orbitals on each of the Mn ions and the O5O6 magnetic orbital. The representation also demonstrates clearly the σ_2p_* nature of the magnetic orbital for O5O6 and the nature of its bonding with the Mn ions of the complex. This orbital interacts in a σ-bonding fashion with the Mn4 ion d-orbitals and has π-bonding interactions with the d-orbitals of Mn3 and Mn1. No significant overlap is found for the Mn2 ion.

**Scheme 1:**
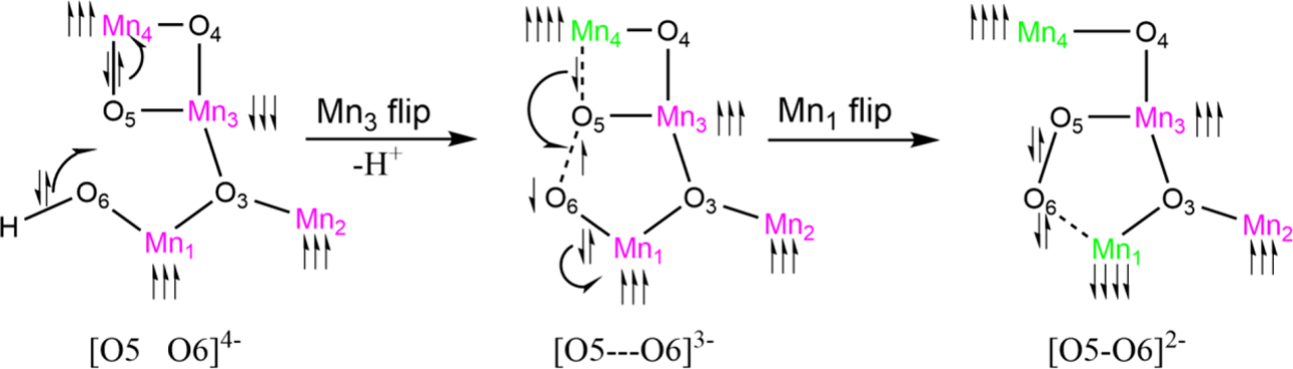
Schematic Electron Flow Pattern Based on Our PES and IBO Analyses from Figures 3 and 4. O5–O6 Oxidation Status Indicated

Our PES and IBO analyses therefore show that low-barrier O–O bond formation is facilitated in the WOC by providing a concerted flow of electrons between the coupling oxo’s, O5 and O6, with the Mn_1_ and Mn_4_ ions providing low-barrier spin crossovers to occur. Scheme 1 demonstrates the key electron movements and spin flips involved. Figure S5 uses a simple molecular orbital scheme to illustrate the species involved with the concerted flow of electron from the σ_2p_* orbital to Mn_1_ and Mn_4_ during the O–O bond formation, permitting the formation of the O–O bond without double occupation of the high-energy orbital.

### Crystallographic and Electron Paramagnetic Resonance Analysis

Studies using X-ray free electron laser (XFEL) atomic resolution structures of the 2-flash state, predominantly S_3_ state, generally support the participation of O5 and O6 in O–O bond formation. Suga et al.^20^ first reported an O5–O6 bond distance of 1.5 Å, indicating peroxo formation.^8,21^ Later studies proposed an additional oxygen O_*x*_ similar to O6 of the structure reported by Suga et al. but with an extended O5–O6/O_*x*_ bond length of 2.1 Å.^11,22,23^ More recently, Suga et al.^18^ proposed a best fit O5–O6 bond length of 1.9 Å. All structures of S_3_ so far appear to rule out an oxo–hydroxo nonbonded form which requires an O5–O6 bond separation of at least 2.5 Å. Additional structural features are a relatively long Mn4–O5 bond length of 2.2 Å and a short Mn1–O6 bond distance of 1.7 Å. Comparison of our calculated minimum energy Ms = 6 structure with the experimental determinations is given in Table 1. This demonstrates excellent agreement with the minimum energy point of this state and the experimental XFEL values supporting the presence of [O5O6]^3–^. In addition, recent time-resolved structural changes for the 2F state formation show significant increases (0.2-0.4 Å) in the Mn4–W2, Mn4–Glu333, (Mn–O) bond distances at the 150 μs time point.^23^ Both would indicate a reduction of Mn4 from IV to III, supporting the formation of [O5O6]^3–^.

**Table 1:** Comparison of Key Calculated Minimum Energy Structure Bond Distances (Å) and Experimental XFEL determinations^a^

	O5–O6	Mn_4_–O5	Mn_3_–O5	Mn_1_–O6	Mn_4_–Mn_3_	Mn_3_–Mn_2_	Mn_2_–Mn_1_	Mn_1_–Mn_3_	Mn_4_–Mn_1_
[O5O6]^3–^	2.0	2.1	1.8	1.7	2.8	2.8	2.8	3.4	5.2
Kern 2018^11^	2.1	2.2	2.0	1.8	2.8	2.9	2.8	3.3	5.1
Suga 2019^18^	1.9	2.2	1.9	1.7	3.0	2.7	2.5	3.4	5.3

a XFEL bond distances reported are an average from both a and a chains of the deposited crystal structures.

**Figure 7:**
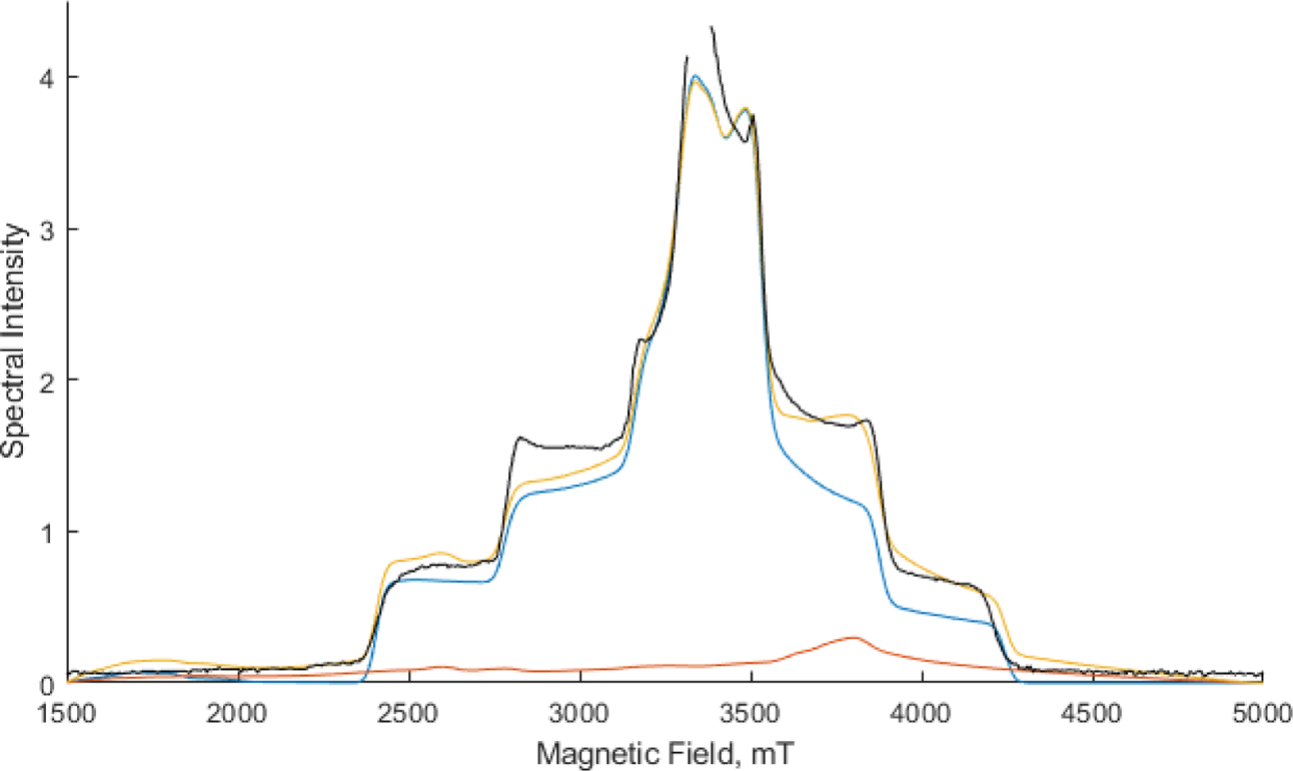
Comparison of 94 GHz EPR spectral simulations of *S* = 6 (red), *S* = 3(blue), and a 0.7:0.3 mixture of *S* = 6:*S* = 3 (yellow). The experimental spectrum from Chrysina et al.^13^ is shown in black. Simulation parameters used are *S* = 6, *g* = 2, *D* = 1.523 cm^–1^, *E*/*D* = 0.14 and S = 3, *g* = 2, D = 0.179 cm^–1^, *E*/*D* = 0.28.

The interpretation of the *S* = 3 signal EPR from the 2-flash state based on the BS-DFT analysis of the calculated hfcs is highly indicative of an oxo–hydroxo form for the S_3_ state.^10,24,25^ An oxo–hydroxo model is not however compatible with the structure obtained by XFEL. As described above, the [O5O6]^3–^ model does agree with the XFEL structures. This corresponds to a broken symmetry Ms = 6 spin state. This is not a true spin state, *S*. The true spin state energies can be obtained by the diagonalization of the Heisenberg Dirac van Vleck Hamiltonian using *J* values obtained by analyzing all possible BS states.^26^Table S1 shows the calculated *J* values and energies of the ground spin states using this procedure. From this, an *S* = 6 spin state is calculated to be the ground-state spin. This, therefore, cannot be attributed to the species observed by EPR/EDNMR, which has an *S* = 3 ground-state spin. The PES shows that the two species are related by the protonation state of O6. Intriguingly, an *S* = 6 species was proposed to be formed in the 2-flash S_3_ state of spinach samples and was proposed to be the major component (80%) of native samples.^27^ The *S* = 6 form was attributed to the so-called closed cubane form of the WOC cluster with a penta-coordinated Mn_4_ (IV) ion formed before the second substrate binds. So far, no structural experimental support for such a closed cubane structure of the WOC has been obtained for any *S* state.^22^ It is therefore more likely (see below) that this species corresponds to the [O5O6]^3–^ form alluded to in this manuscript, also with *S* = 6. Experimentally, no *S* = 6 species has so far been reported in cyanobacteria samples, where the high-resolution high-field W-band EPR spectra obtained are attributed to an *S* = 3 form^13^ Simulations of the W-band EPR spectra for the *S* = 3 form are shown in Figure 7. Also shown are simulations for an *S* = 6 form using the zero-field splitting parameters reported for the spinach samples.^27^ From the simulations, it is clear that the spectral intensity of the S = 6 form is much less than that of the *S* = 3 form. This suggests that the *S* = 6 form would be difficult to detect in the W-band EPR experiment. More intriguingly, as shown in Figure 7, even with a 70% contribution of the *S* = 6 form, the *S* = 3 form still dominates the spectral envelope, with the *S* = 6 form mainly contributing a distinctive shoulder at around 3500–4000 mT to the overall spectral shape.

It is clear from the spectra presented in Figure 3 of Chrysina et al.^13^ that a poorer fit between the experimental and a simulated *S* = 3 spectrum exists in this very region. Figure 7 shows that the inclusion of the *S* = 6 form (70%) gives rise to a much improved fit to the experimental spectrum. Additional simulations performed by varying the ratio of the two spin systems are presented in Figures S7 where we can estimate that an *S* = 6 contribution between 60 and 70% is optimal. We therefore suggest that the seeming incompatibility between the XFEL and EPR data for the S_3_ state lies in the fact that the oxo–hydroxo and [O_2_]^3–^ forms are in equilibrium. The [O_2_]^3–^ form detected in the XFEL-determined structure is not readily apparent in the EPR spectrum due to its *S* = 6 nature and the resultant low intensity compared with the *S* = 3 form. Further simulations presented in Figure S7 suggest that the *S* = 6 component is also likely a major component of the broadened W-band EPR spectrum caused by methanol and glycerol addition.^13^ It has been known for some time that the *S* = 3 species does not correspond to all of the S_3_ spin and that an EPR-“undetectable” component observed only on near-infrared (NIR) irradiation is also present in equilibrium with it.^28,29^ In our analysis, this undetectable component corresponds to the [O_2_]^3–^ form. This is a different assignment to that previously made for the *S* = 6 form detected in spinach samples where the *S* = 6 form was attributed to a closed cubane form of the WOC cluster with a penta-coordinated Mn_4_ (IV) ion, an intermediate formed prior to the binding of the second substrate water.^27^ This, however, in striking contrast to the model proposed here, is not supported by the XFEL structural data.^23^ In addition, it has been shown that Mn(III) is required for NIR excitation,^30^ and the large *D* value of 1.523 cm^–1^ for the *S* = 6 form strongly suggests the presence of Mn(III) ion in the complex. It should be noted that it is possible that the peroxo form, Figure 2, is also present in a low concentration, and its EPR spectrum is masked by the oxo–hydroxo form. The peroxo complex would have two Mn(III) ions present, likely leading to a large *D* value similar to the [O_2_]^3–^ form which would again lead to a low-intensity EPR spectrum compared with the oxo–hydroxo form.

### X-ray Emission Spectroscopy Analysis

Further experimental support for our S_3_ state model comes from the analysis of the X-ray emission spectroscopy (XES) data by Ibrahim et al.^23^ The 1F flash first moment XES shift can be confidently assigned to Mn (III) to Mn (IV) oxidation of Mn_4_. The first moment shift for the 2F state is approximately 40% of the 1F shift based on the solution-phase data^23^ and the most current time-resolved crystal data (see Figure S9). In addition, at least 10% of the oxidation change shift can be attributed to S_1_-to-S_2_ oxidation based on the S state populations of the 1F state reported by Ibrahim et al.,^23^ leaving around 30% Mn oxidation occurring in the S_2_-to-S_3_ transition. This is what is predicted by our equilibrium model above. The 30% Mn oxidation can be attributed to the formation of the oxo–hydroxo form where Mn_1_(III)-to-Mn_1_(IV) oxidation occurs. The [O5O6]^3-^ form has, however, an overall Mn oxidation state identical to the S_2_ state, that is, one Mn(III) and three Mn(IV), so this will not give rise to a first moment shift.

**Figure 8:**
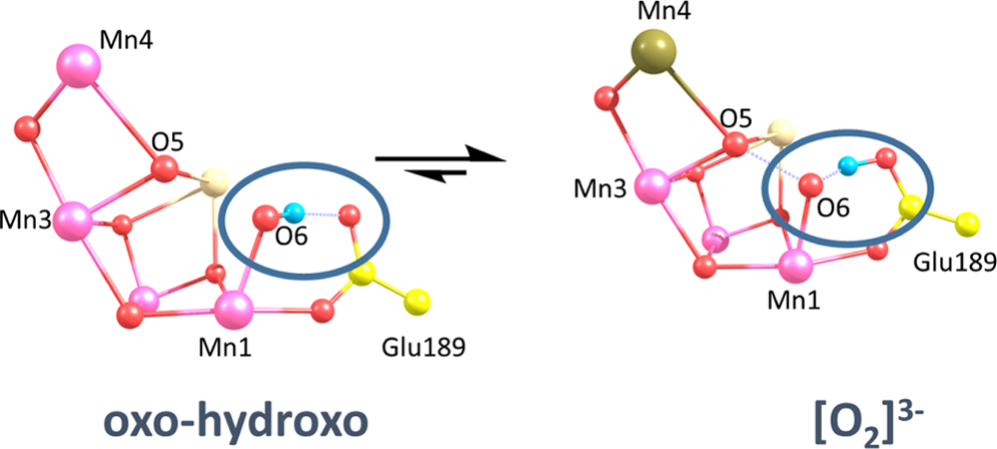
S_3_ state equilibrium between oxo–hydroxo and [O_2_]^3–^, highlighting the proposed proton shuffle between O6 and Glu189. Gold color, Mn(III); purple, Mn(IV).

The computational, structural, and spectroscopic evidence above all points to an S_3_ state involving an equilibrium between an O5–O6H oxo–hydroxo and an [O5O6]^3–^ species. The most recent XFEL structures for the S_3_^22,23^ state also reveal a very short O6 to OEGlu189 distance of 2.4–2.5 Å, suggesting a low-barrier hydrogen bond between the two atoms. This strongly indicates that the S_3_ state equilibrium is established by proton-sharing between these two atoms, as illustrated in Figure 8.

**Figure 9:**
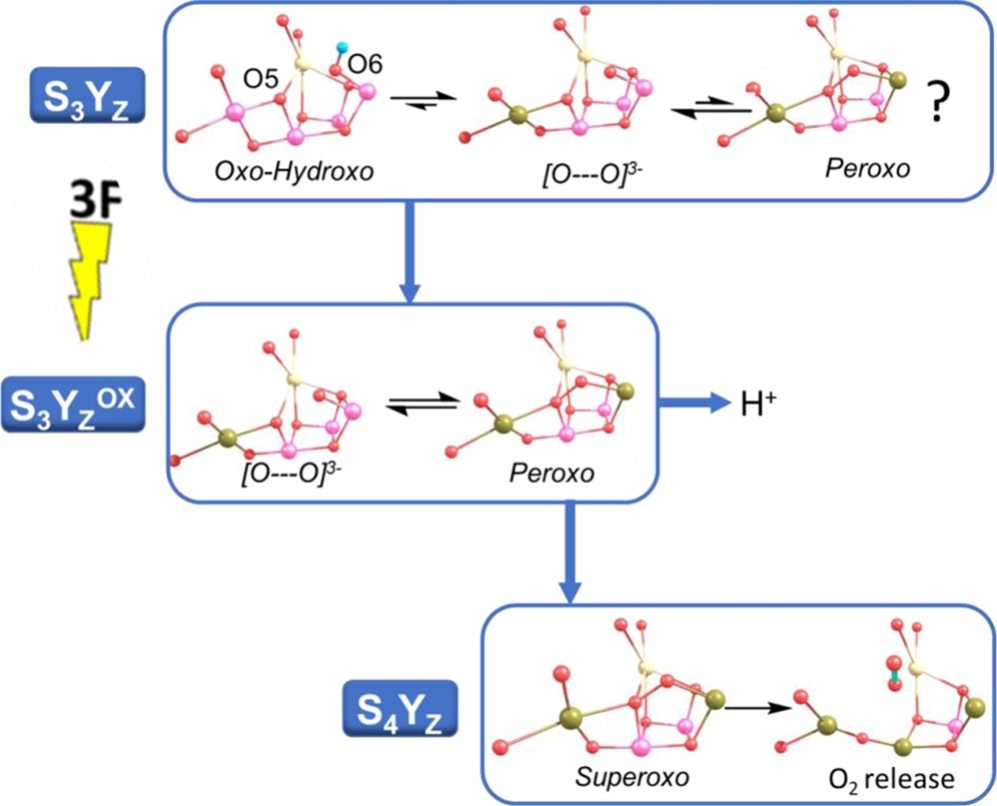
Proposed O_2_ formation mechanism for the WOC. Gold color, Mn(III); purple, Mn(IV). See text for details.

Based on our combined computational, spectroscopic, and structural analysis, we demonstrate that O–O bond formation has begun between the O5 and O6 atoms in the S_3_ state, with the generation of the [O5O6]^3–^ ion. This is the dominant species present in the S_3_ state. Figure 2 shows that this provides a low-barrier pathway to the subsequent formation of the peroxo form. As indicated above, this peroxo

form could be present in a low concentration in the S_3_ state and may be further stabilized after the fourth flash on generation of the S_3_ Y_Z_^OX^ state and further removal of a proton from the WOC, Figure 9.

Subsequent oxidation of the WOC by Y_Z_^OX^ leads to the oxidation of peroxo, leading to the transient superoxo formation which will rapidly lead to triplet O_2_ formation and release from the WOC.^8^ The initiation of O–O bond formation in the S_3_ or S_3_Y_Z_^OX^ state is supported by kinetic findings which have shown that there is a kinetic coincidence between the rate of O_2_ evolution and Y_Z_^OX^ reduction.^31^ Time-resolved X-ray emission studies^32^ have demonstrated that reduction as opposed to oxidation of the WOC occurs after the third flash, fully supporting O–O bond formation in the S_3_ and S_3_Y_Z_^OX^ states.

## Conclusions

Analysis of the electronic structure changes along the reaction path for the O5–O6 bond formation in the S_3_ state of the WOC shows that two spin crossovers, facilitated by the geometry and magnetism of the water-oxidizing complex, are used to provide a unique low-energy pathway. The pathway is facilitated via formation and stabilization of the [O5O6]^3-^ ion. This [O_2_]^3–^ ion is stabilized by antiferromagnetic interaction with the Mn ions of the complex. The combined computational, crystallographic, and spectroscopic data show that an equilibrium exists between an O5 oxo and an O6 hydroxo form, *S* = 3 spin state, and a deprotonated O6 form containing a two-center one-electron bond in [O5O6]^3–^ which we identify as the form detected by XFEL crystallography. This form gives rise to an *S* = 6 spin state which gives rise to a low-intensity EPR spectrum compared with the accompanying *S* = 3 state, making its detection via EPR difficult and overshadowed by the *S* = 3 form. Simulations assuming a 70% contribution of the *S* = 6 form give rise to a superior fit to the experimental EPR spectrum compared with an *S* = 3 only form. The study reveals the key electronic, magnetic, and structural design features of nature’s catalyst, which allows water oxidation to O_2_ to be uniquely performed under ambient conditions.

## Supporting Information Available

Details of calculations performed and models used and additional data supporting the main text (PDF)
