# Supplementary material for: How Nature Makes O2: an Electronic Level Mechanism for Water Oxidation in Photosynthesis
Source: J Phys Chem B. 2022 Oct 7;126(41):8214–21. doi: 10.1021/acs.jpcb.2c06374 (PMC9589598; doi:10.1021/acs.jpcb.2c06374)
Supplement: Supplementary file 1 — jp2c06374_si_001.pdf [file jp2c06374_si_001.pdf]

How Nature makes O_2: an electronic level mechanism for water oxidation in photosynthesis.

Felix Rummel and Patrick J O'Malley*,

Department of Chemistry, School of Natural Sciences, The University of Manchester, Manchester, M13 9PL, UK.

E-mail: patrick.omalley@manchester.ac.uk

Methods

The computational procedure used is similar to those described previously.[1,2] All calculations were performed using ORCA 4[3] with models initially geometry optimised in the HS oxo-oxo oxidation state of $(Mn^{III})_4$.[2] All calculations used the B3LYP functional[4,5] with the zeroth-order regular approximation (ZORA) Hamiltonian applied to include scalar relativistic effects.[6–8] ZORA versions of the def2-SVP basis sets were used for C and H atoms. ZORA versions of the def2-TZVP basis set with f functions removed were used for all other atoms.[9] The B3LYP functional was chosen as appropriate for a system of the size studied and because it has a proven track record for energetic and orbital analysis of reactions of this type [10] providing accurate energetic results, particularly for the WOC [11] and many other challenging transition metal based systems.[12] The chain of spheres (RIJCOSX) approximation to exact exchange was applied along with the decontracted general Weigend auxiliary basis sets.[13–17] Dispersion corrections proposed by Grimme with Becke-Johnson damping (D3BJ) were included.[18,19] The conductor-like polarizable continuum model (CPCM) with a dielectric constant $\varepsilon = 8.0$[20,21] was applied in all calculations. Increased integration grids (Grid6 and IntAcc 6 in ORCA convention) and tight SCF convergence

criteria were used with all terminal carbon atoms constrained during optimisation calculations.

To calculate initial BS-DFT wavefunctions for potential energy surface calculations, ZORA versions of the def2-TZVP basis set with f functions removed were used for all atoms.[9] Initial BS guesses were constructured using the 'flipspin' feature of ORCA.[22] Convergence to the correct BS and HS states in all calculations were confirmed by examination of the calculated Mulliken spin populations.

The potential energy surface was calculated using the geometry optimisation method described above, initially reading in the broken symmetry ".gbw" file to correctly model the M_s=3 state. The O5-O6 bond length was varied from 2.44 to 1.44 Å in several steps with full geometry optimisation at each step to produce the full energy surface in the text. Intrinsic bond orbitals (IBOs) were produced from the optimised PES calculation wavefunctions using IboView with iboexp=2.[23] To obtain the corresponding orbitals illustrating the overlap between the Mn ions and the O5O6 magnetic orbital σ2p*, we use an atom substitution approach to "switch off" pathways except the interaction between individual Mn ions and the O5O6 orbital. This was achieved by substituting the other ions with either Ga (position 4) or Ge (positions 1, 2 and 3).

All EPR spectral simulations have been performed using the EasySpin 5.2.35 'pepper' function within MATLAB. EPR parameters are given in the text and figure captions.

Model systems

All models were generated from the S_3 XFEL crystal structure (PDB: 6DHO)[24] and geometry optimised into the S_3 state. Seven directly coordinated amino acids are included in the models. Six are from the D1 protein chain (Asp-170, Glu-189, His-332, Glu-333, Asp-342, Ala-344) and one from the CP43 protein chain (Glu-354). The second sphere His-337 residue

was included along with the partial backbone of Glu-329. All terminal carbon atoms were constrained throughout the calculations. The models (Figure S1 and S2) contain four directly coordinated water molecules, two bound to Mn_4 (W1 and W2) and two bound to Ca (W3 and W4), along with ten crystallographic water molecules. All bridging oxygen atoms are in the μ-oxo (O^{2-}) form and all coordinated water ligands W1-W4 are in the aquo form.

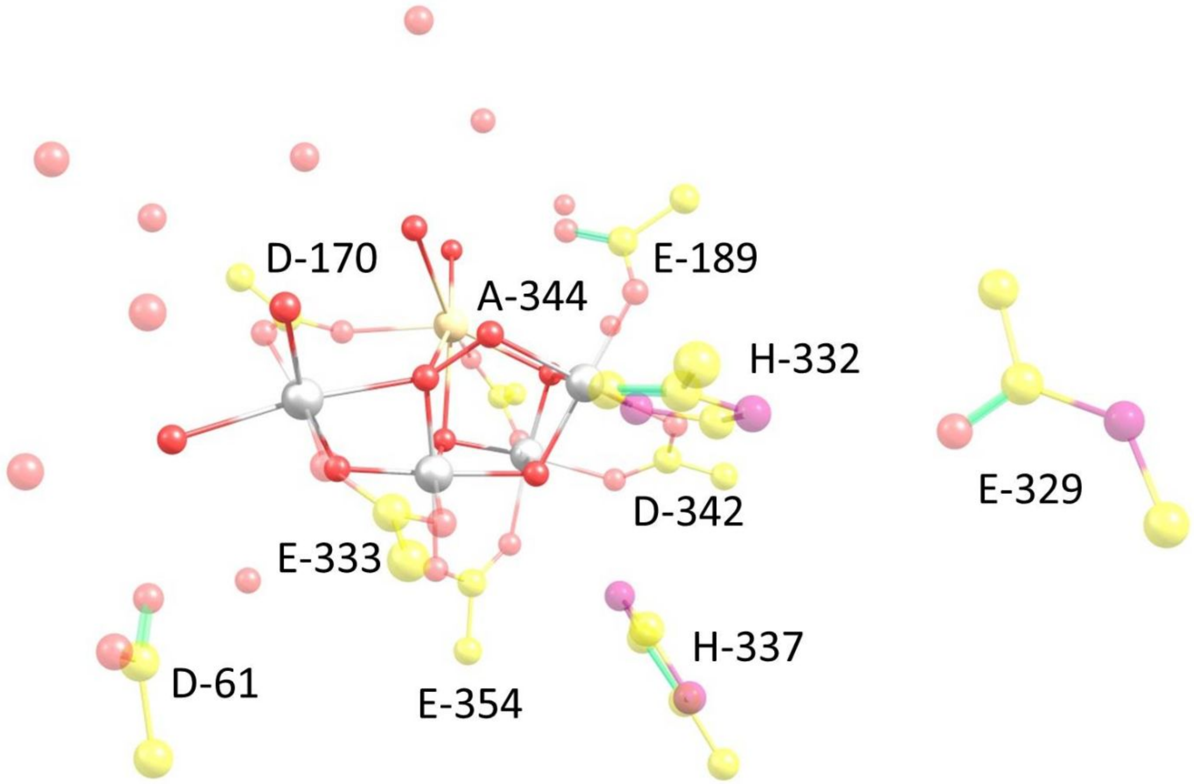

Figure S1 Model used as the starting point for all PES calculations. Hydrogens have been excluded for clarity. Colour coding: Mn (silver), oxygen (red), calcium (white), carbon (yellow) and nitrogen (purple).

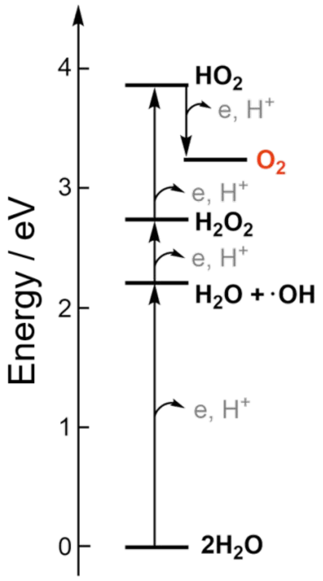

Figure S2. . Energy level diagram for four sequential electron oxidations of water to O2 in the aqueous phase.

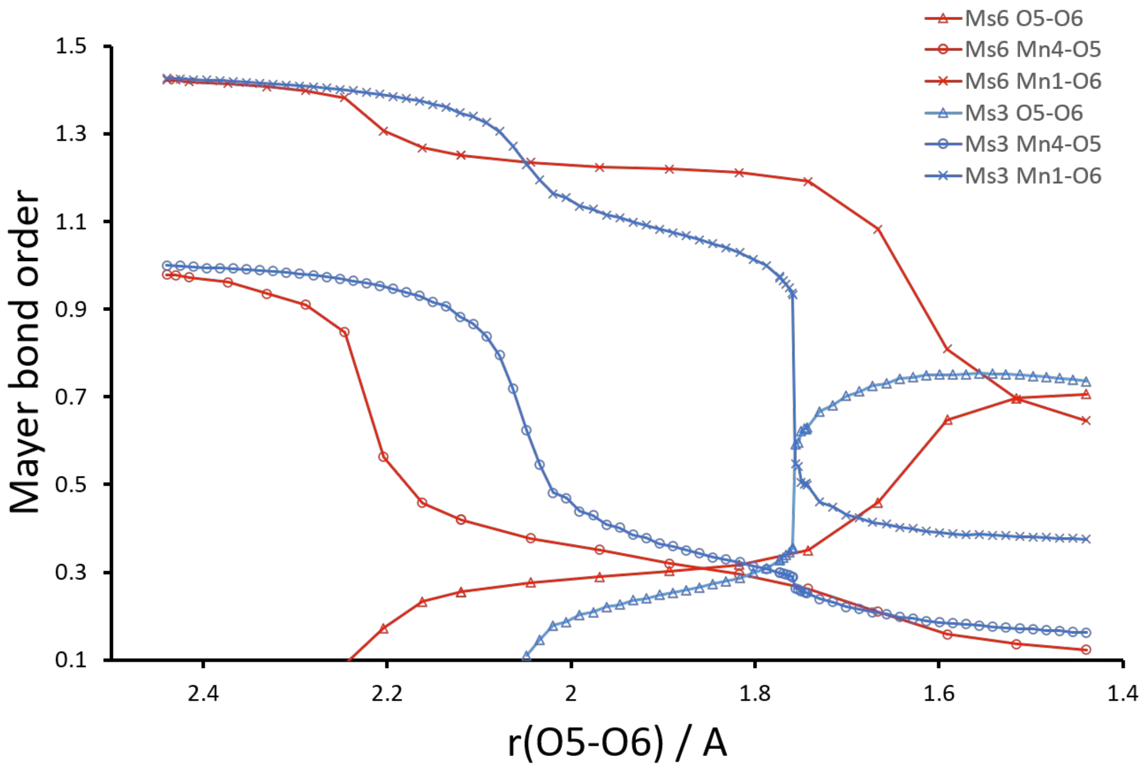

Figure S3 . Variation of Mayer bond orders for the oxo-oxo Ms=6 and Ms=3 BS states along the O5O6 PES.

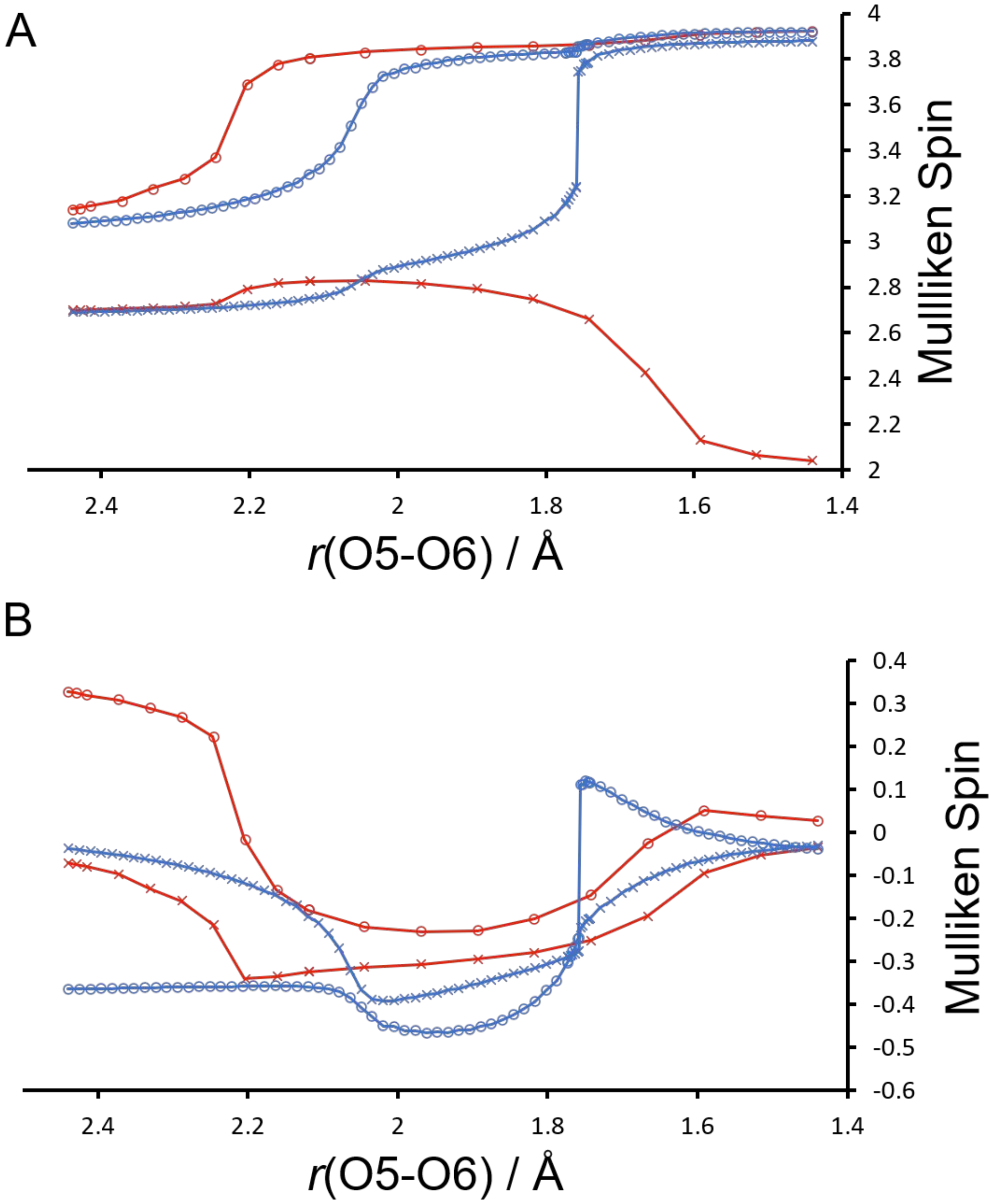

Figure S4. Variation of Mulliken spin populations along the O5O6 PES for oxo-oxo Ms=3 (blue) and Ms=6 (red). A. Mn1 (x) and Mn4 (circle). B. O5 (x) and O6 (circle)

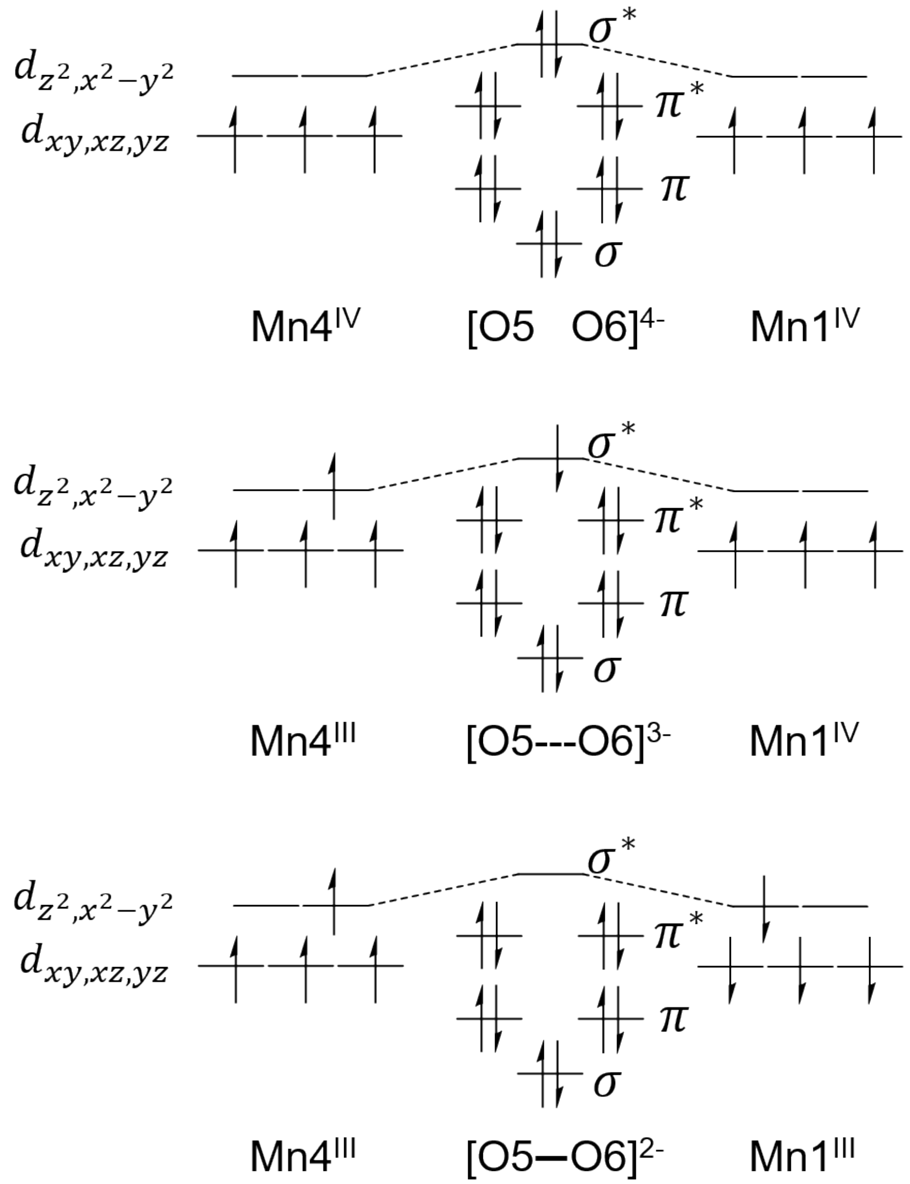

Figure S5. Molecular orbital schematic of electronic rearrangements leading to O-O bond formation. For the oxo-hydroxo form, the bond order of zero for $[O5O6]^{4-}$ signifies no covalent bond can exist between O5 and O6. One electron transfer from the σ2p* orbital to Mn4 leads to formation of the $[O5O6]^{3-}$ species and a situation with doubly occupied σ2p and one-electron (β) occupied σ2p* orbitals, resulting in an O5O6 bond order of 0.5. Transfer of the remaining σ2p* electron to Mn1 results in the formation of an O5-O6 single bond (peroxo) with a bond order of 1. The key to low-barrier O-O formation in the WOC is the sequential removal of two electrons from the combining O6 and O5 oxos by Mn1 and

Mn4 respectively thereby resulting in an unoccupied σ_{2p}* orbital and low barrier O-O bond formation.

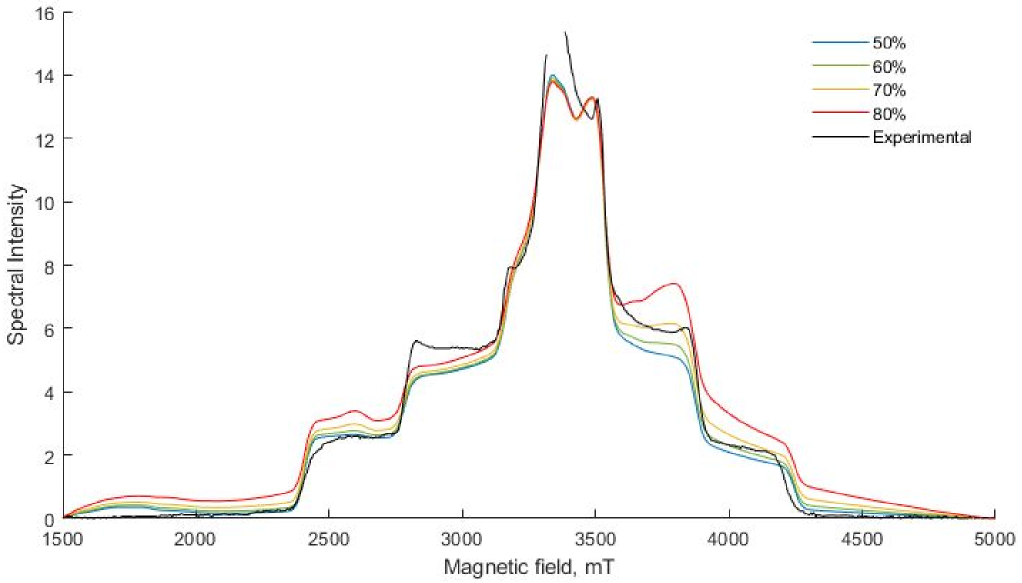

Figure S6. Comparison of best simulation fit with experimental W-band EPR spectrum (black) using various percentages of the S=6 signal. Parameters used as given in Figure 7 of the main text.

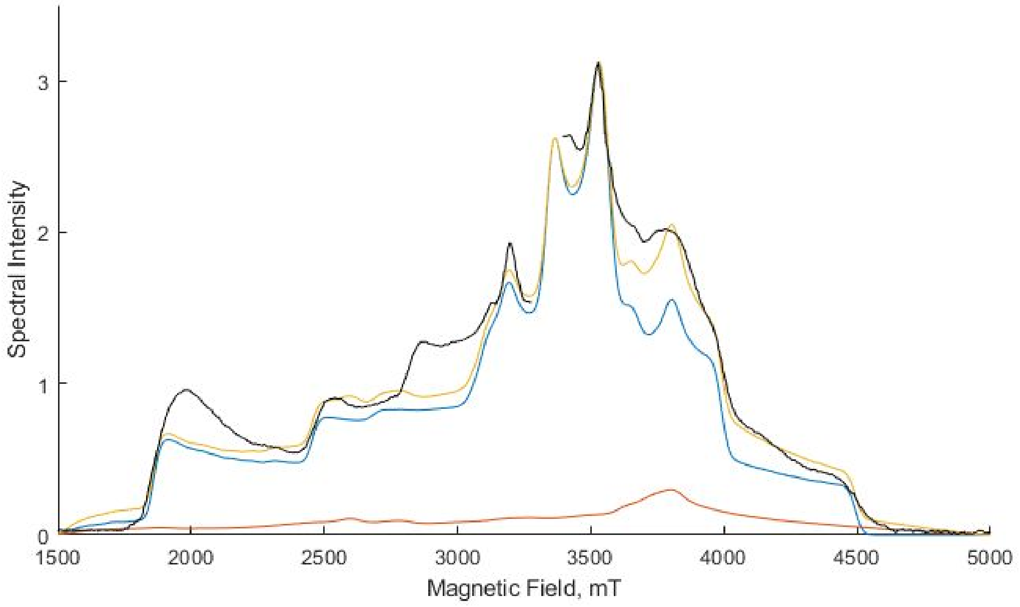

Figure S7. Simulated W-band EPR spectrum for broadened S3 state signal observed after methanol treatment. Red, S=6, D = 1.523 cm^{-1} E/D = 0.14 ; blue S=3, D = 0.281 cm^{-1}, E/D = 0.16; yellow 70% of S=6. Black experimental spectrum . As for the native sample described in main text, the fit to experimental spectrum in the region 3500 – 4500 mT is much improved with the inclusion of an S=6 component.

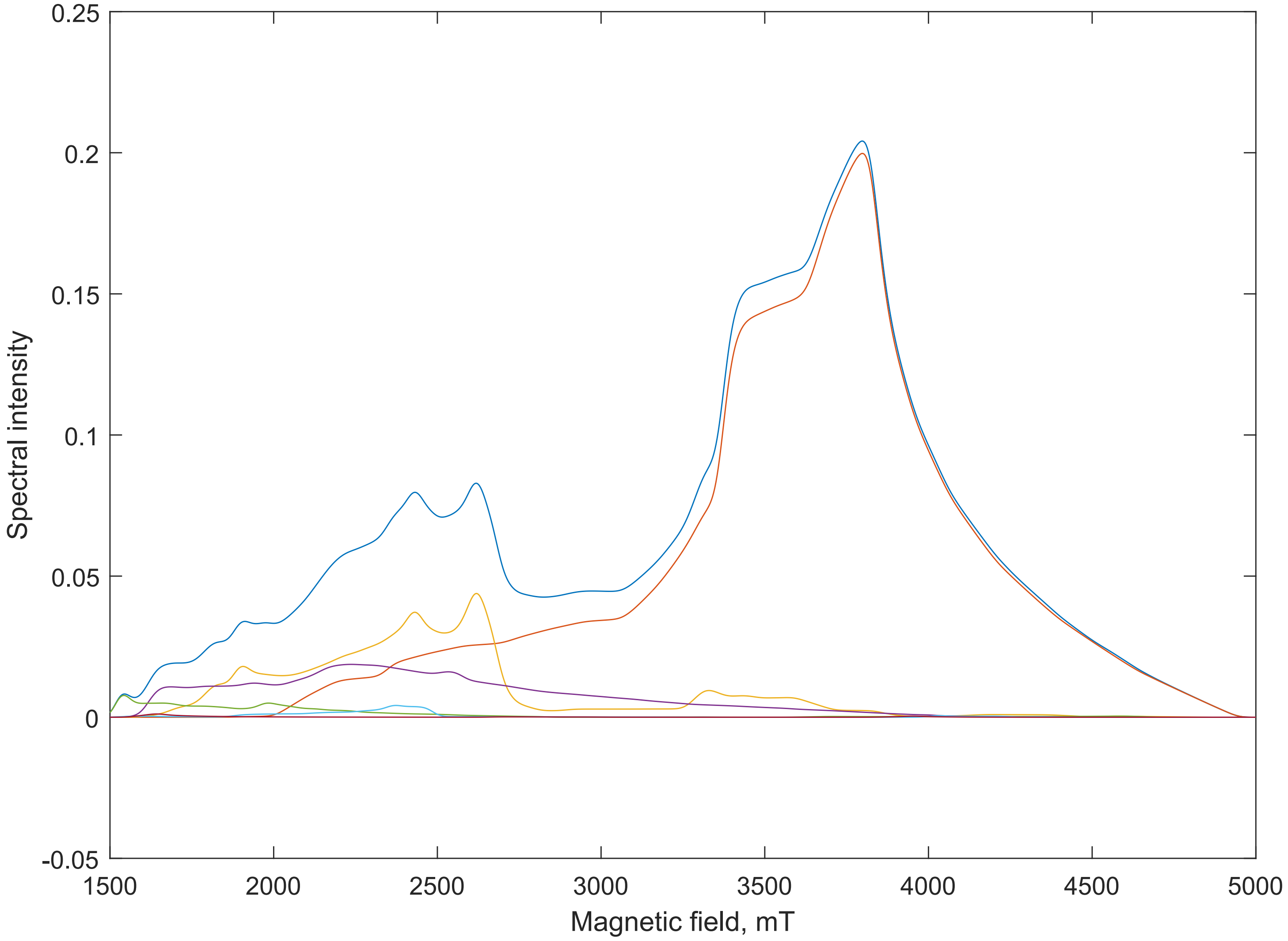

Figure S8. S=6, 94 GHz EPR spectrum simulation. Blue envelope corresponds to overall spectrum with the decomposition of this overall envelope into individual transitions shown below, see also Figure S8. Simulation parameters, g=2, D =1.523 cm^{-1} and E/D =0.14.

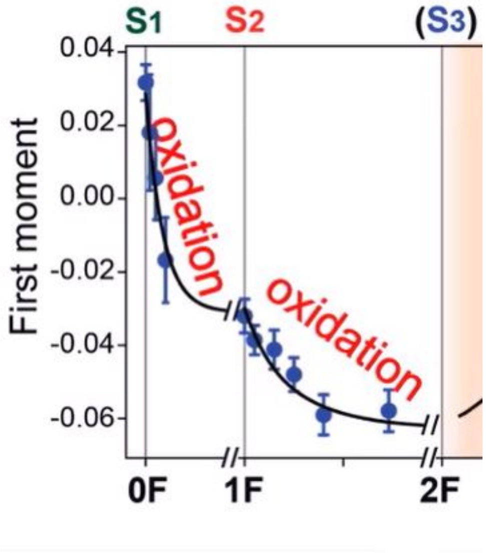

Figure S9. Time resolved XES first moment data for the 1F and 2F transitions reproduced from Yano, Y., International Congress on Photosynthesis Research , August 2022, Dunedin New Zealand. The data show an approximately 40% magnitude oxidation change for 2F compared with 1F. This is fully in line with an equilibrium model for the S_3 state comprising oxo-hydroxo (30-40%) and $[O_2]^{3-}$ (60-70%) as discussed in main text.

Table S1: BS-DFT calculated Mn_i/Mn_j exchange couplings (J_{ij}, cm^{-1}). HDvV calculated total spin (S) ground state (GS) and first excited state (ES) and separation energies ($\Delta E, cm^{-1}$) for $[O5O6]^{3-}$.

	$[O5O6]^{3-}$
J_{43}	-18
J_{42}	1
J_{41}	-88
$J_{4\text{-}O6/O5}$	-1052
J_{32}	11
J_{31}	-26
$J_{3\text{-}O6/O5}$	-433
J_{21}	14
$J_{2\text{-}O6/O5}$	0
$J_{1\text{-}O6/O5}$	-1301
S_{GS}	6
S_{ES}	5
ΔE	116.7

References

(1) Corry, T. A.; O'Malley, P. J. Proton Isomers Rationalize the High- and Low-Spin Forms of the S2 State Intermediate in the Water-Oxidizing Reaction of Photosystem II. *J. Phys. Chem. Lett.* **2019**, *10* (17), 5226–5230.

(2) Corry, T. A.; O'Malley, P. J. Evidence of O-O Bond Formation in the Final Metastable S3 State of Nature's Water Oxidizing Complex Implying a Novel Mechanism of Water Oxidation. *J. Phys. Chem. Lett.* **2018**, *9* (21), 6269–6274.

(3) Neese, F. Software Update: The ORCA Program System, Version 4.0. *Wiley Interdiscip. Rev. Comput. Mol. Sci.* **2018**, *8* (1), e1327.

(4) Lee, C.; Yang, W.; Parr, R. G. Development of the Colle-Salvetti Correlation-Energy Formula into a Functional of the Electron Density. *Phys. Rev. B* **1988**, *37* (2), 785–789.

(5) Becke, A. D. A New Mixing of Hartree-Fock and Local Density-Functional Theories. *J. Chem. Phys.* **1993**, *98* (2), 1372–1377.

(6) Lenthe, E. Van; Baerends, E. J.; Snijders, J. G. Relativistic Regular Two-Component Hamiltonians. *J. Chem. Phys.* **1993**, *99* (6), 4597.

(7) van Lenthe, E.; Baerends, E. J.; Snijders, J. G. Relativistic Total Energy Using Regular Approximations. *J. Chem. Phys.* **1994**, *101* (11), 9783.

(8) van Wüllen, C. Molecular Density Functional Calculations in the Regular Relativistic Approximation: Method, Application to Coinage Metal Diatomics, Hydrides, Fluorides and Chlorides, and Comparison with First-Order Relativistic Calculations. *J. Chem. Phys.* **1998**, *109* (2), 392.

(9) Weigend, F.; Ahlrichs, R. Balanced Basis Sets of Split Valence, Triple Zeta Valence and Quadruple Zeta Valence Quality for H to Rn: Design and Assessment of Accuracy. *Phys. Chem. Chem. Phys.* **2005**, *7* (18), 3297.

(10) Klein, J. E. M. N.; Knizia, G. CPCET versus HAT: A Direct Theoretical Method for Distinguishing X–H Bond-Activation Mechanisms. *Angew. Chemie - Int. Ed.* **2018**, *57* (37), 11913–11917.

(11) Siegbahn, P. E. M. Nucleophilic Water Attack Is Not a Possible Mechanism for O–O Bond Formation in Photosystem II. *Proc. Natl. Acad. Sci.* **2017**, *114* (19).

(12) Altun, A.; Breidung, J.; Neese, F.; Thiel, W. Correlated Ab Initio and Density Functional Studies on H2activation by FeO+. *J. Chem. Theory Comput.* **2014**, *10* (9), 3807–3820.

(13) Eichkorn, K.; Treutler, O.; Öhm, H.; Häser, M.; Ahlrichs, R. Auxiliary Basis Sets to Approximate Coulomb Potentials. *Chem. Phys. Lett.* **1995**, *240* (4), 283–289.

(14) Eichkorn, K.; Weigend, F.; Treutler, O.; Ahlrichs, R. Auxiliary Basis Sets for Main Row Atoms and Transition Metals and Their Use to Approximate Coulomb Potentials. *Theor. Chem. Acc.* **1997**, *97* (1–4), 119–124.

(15) Weigend, F. Accurate Coulomb-Fitting Basis Sets for H to Rn. *Phys. Chem. Chem. Phys.* **2006**, *8* (9), 1057–1065.

(16) Staroverov, V. N.; Scuseria, G. E.; Tao, J.; Perdew, J. P. Comparative Assessment of a New Nonempirical Density Functional: Molecules and Hydrogen-Bonded Complexes. *J. Chem. Phys.* **2003**, *119* (23), 12129.

(17) Neese, F.; Wennmohs, F.; Hansen, A.; Becker, U. Efficient, Approximate and Parallel Hartree-Fock and Hybrid DFT Calculations. A "chain-of-Spheres" Algorithm for the Hartree-Fock Exchange. *Chem. Phys.* **2009**, *356* (1–3), 98–109.

(18) Grimme, S.; Antony, J.; Ehrlich, S.; Krieg, H. A Consistent and Accurate Ab Initio Parametrization of Density Functional Dispersion Correction (DFT-D) for the 94 Elements H-Pu. *J. Chem. Phys.* **2010**, *132* (15), 154104.

(19) Grimme, S.; Ehrlich, S.; Goerigk, L. Effect of the Damping Function in Dispersion Corrected Density Functional Theory. *J. Comput. Chem.* **2011**, *32* (7), 1456–1465.

(20) Cox, N.; Retegan, M.; Neese, F.; Pantazis, D. A.; Boussac, A.; Lubitz, W. Photosynthesis. Electronic Structure of the Oxygen-Evolving Complex in Photosystem II Prior to O-O Bond Formation. *Science* **2014**, *345* (6198), 804–808.

(21) Pantazis, D. A.; Ames, W.; Cox, N.; Lubitz, W.; Neese, F. Two Interconvertible Structures That Explain the Spectroscopic Properties of the Oxygen-Evolving Complex of Photosystem II in the S2 State. *Angew. Chem. Int. Ed. Engl.* **2012**, *51* (39), 9935–9940.

(22) Neese, F. The ORCA Program System. *Wiley Interdiscip. Rev. Comput. Mol. Sci.* **2012**, *2* (1), 73–78.

(23) Knizia, G.; Klein, J. E. M. N. Electron Flow in Reaction Mechanisms - Revealed from First Principles. *Angew. Chemie - Int. Ed.* **2015**, *54* (18), 5518–5522.
